# Stimulating VAPB-PTPIP51 ER-mitochondria tethering corrects FTD/ALS mutant TDP43 linked Ca^2+^ and synaptic defects

**DOI:** 10.1186/s40478-024-01742-x

**Published:** 2024-02-23

**Authors:** Andrea Markovinovic, Sandra M. Martín-Guerrero, Gábor M. Mórotz, Shaakir Salam, Patricia Gomez-Suaga, Sebastien Paillusson, Jenny Greig, Younbok Lee, Jacqueline C. Mitchell, Wendy Noble, Christopher C.J. Miller

**Affiliations:** Department of Basic and Clinical Neuroscience, Institute of Psychiatry, Psychology and Neuroscience, King’s College London, SE5 9RX London, UK

**Keywords:** Neurodegenerative diseases, Frontotemporal dementia, Amyotrophic lateral sclerosis, TDP43, Alzheimer’s disease, Parkinson’s disease

## Abstract

Frontotemporal dementia (FTD) and amyotrophic lateral sclerosis (ALS) are clinically linked major neurodegenerative diseases. Notably, TAR DNA-binding protein-43 (TDP43) accumulations are hallmark pathologies of FTD/ALS and mutations in the gene encoding TDP43 cause familial FTD/ALS. There are no cures for FTD/ALS. FTD/ALS display damage to a broad range of physiological functions, many of which are regulated by signaling between the endoplasmic reticulum (ER) and mitochondria. This signaling is mediated by the VAPB-PTPIP51 tethering proteins that serve to recruit regions of ER to the mitochondrial surface so as to facilitate inter-organelle communications. Several studies have now shown that disrupted ER-mitochondria signaling including breaking of the VAPB-PTPIP51 tethers are features of FTD/ALS and that for TDP43 and other familial genetic FTD/ALS insults, this involves activation of glycogen kinase-3β (GSK3β). Such findings have prompted suggestions that correcting damage to ER-mitochondria signaling and the VAPB-PTPIP51 interaction may be broadly therapeutic. Here we provide evidence to support this notion. We show that overexpression of VAPB or PTPIP51 to enhance ER-mitochondria signaling corrects mutant TDP43 induced damage to inositol 1,4,5-trisphosphate (IP3) receptor delivery of Ca^2+^ to mitochondria which is a primary function of the VAPB-PTPIP51 tethers, and to synaptic function. Moreover, we show that ursodeoxycholic acid (UDCA), an FDA approved drug linked to FTD/ALS and other neurodegenerative diseases therapy and whose precise therapeutic target is unclear, corrects TDP43 linked damage to the VAPB-PTPIP51 interaction. We also show that this effect involves inhibition of TDP43 mediated activation of GSK3β. Thus, correcting damage to the VAPB-PTPIP51 tethers may have therapeutic value for FTD/ALS and other age-related neurodegenerative diseases.

## Introduction

Frontotemporal dementia (FTD) is the second most common form of presenile dementia after Alzheimer’s disease and amyotrophic lateral sclerosis (ALS) is the most common form of motor neuron disease. Although originally regarded as distinct diseases, FTD and ALS are now known to form a continuum with significant proportions of FTD patients displaying clinical features of ALS, and ALS patients showing FTD like symptoms [30]. Aside from this clinical overlap, FTD and ALS also display pathogenic similarities. Thus, mutations in the same genes including *TARDBP* (encoding TDP43), *FUS* and *C9orf72* cause dominantly inherited familial forms of FTD and ALS, and abnormal accumulations of TDP43 in affected neurons are hallmark pathologies of both diseases [30].

There are no cures for either FTD or ALS. Many current therapeutic approaches involve correcting damaged physiological processes but the broad range of damage seen in FTD/ALS renders this complex. Thus, damage to mitochondria, the ER including activation of the unfolded protein response, Ca^2+^ signaling, lipid metabolism, autophagy, axonal transport, synaptic function and inflammatory responses are all features of FTD/ALS [32, 33, 39]. The biological conundrum is how so many diverse cellular functions are collectively damaged; the therapeutic challenge is selecting which of these damaged functions to prioritise in drug discovery programmes.

Recently, some attention has focussed on signaling between the ER and mitochondria in FTD/ALS [32, 33, 39]. ER membranes form close contacts with mitochondria and this enables the two organelles to communicate with each other and so respond in an orchestrated fashion to changes in cellular physiology. These regions of ER are termed mitochondria-associated ER membranes. The two primary functions of ER-mitochondria signaling are to synthesise some major phospholipids and to facilitate IP3 receptor delivery of Ca^2+^ from ER stores to mitochondria. This delivery is essential for mitochondrial ATP production since dehydrogenases in the tricarboxylic acid cycle are Ca^2+^-dependent. ER-mitochondria signaling thus regulates both lipid and bioenergetic linked functions such as synaptic activity, autophagy, ER stress and axonal transport, all functions that are damaged in FTD/ALS [6, 32, 39]. A number of studies have now shown that several FTD/ALS linked familial genetic insults disrupt ER-mitochondria communications. These include mutant *TARDBP*, *FUS*, *C9orf72, MAPT* (encoding Tau), *SIGMAR1* (encoding the Sigma1 receptor) and *SOD1* (encoding Cu/Zn superoxide dismutase-1) [3, 8, 7, 18, 20, 29, 42, 47–50, 57].

The mechanisms by which ER membranes form contacts with mitochondria to permit inter-organelle signaling are not properly understood but it is generally agreed that it involves “tethering proteins” that serve to recruit and scaffold ER in close proximity to mitochondria [6, 32, 39]. One well characterised tether involves an interaction between the integral ER protein vesicle-associated membrane protein-associated protein B (VAPB) and the outer mitochondrial membrane protein, protein tyrosine phosphatase interacting protein 51 (PTPIP51) (also known as regulator of microtubule dynamics-3 and family with sequence similarity 82 member A2) [10, 47]. The VAPB-PTPIP51 tethers regulate a number of ER-mitochondria signaling functions including IP3 receptor delivery of Ca^2+^ from ER stores to mitochondria, mitochondrial ATP production, autophagy, phospholipid synthesis and synaptic activity [10, 14, 18–17, 40, 43, 47, 48, 58]. Moreover, familial FTD/ALS linked mutant *TARDBP*, *FUS* and *C9orf72* have all been shown to disrupt the VAPB-PTPIP51 interaction [18, 47, 48]. Most recently, the VAPB-PTPIP51 interaction has been shown to be disrupted in affected motor neurons in post-mortem human ALS spinal cord [21].

The VAPB-PTPIP51 tethering proteins thus regulate many of the diverse functions that are perturbed in FTD/ALS and are themselves disrupted by at least three disease associated genetic insults. Correcting disrupted VAPB-PTPIP51 tethering therefore represents a novel target for therapy and one that may positively impact on a number of downstream functions that are damaged in FTD/ALS. However, at present there is little evidence to support this notion.

Here we address this issue by investigating how enhancing ER-mitochondria signaling by expression of VAPB or PTPIP51 affects TDP43 induced damage to ER-mitochondria Ca^2+^ exchange and associated synaptic defects. We chose to study TDP43 since abnormal TDP43 accumulations are a hallmark pathology of FTD/ALS and because mutations in *TARDBP* cause dominantly inherited familial forms of FTD/ALS; defective TDP43 metabolism is therefore believed to be central to most forms of FTD/ALS [30, 51]. Expression of both wild-type and familial FTD/ALS mutants of TDP43 have been shown to disrupt the VAPB-PTPIP51 interaction, reduce ER-mitochondria contacts, inhibit IP3 receptor delivery of Ca^2+^ to mitochondria and to activate GSK3β to similar extents [47]. This is in line with the phenotypes seen in transgenic mice expressing wild-type or FTD/ALS mutant TDP43 which exhibit similar aggressive disease onset and progression [52]. Such findings reinforce the role of VAPB-PTPIP51 tethering in FTD/ALS; clearly, if only mutant TDP43 disrupted the VAPB-PTPIP51 tethers it would mean that disease seen in wild-type TDP43 mice could not involve damage to the tethers.

We show that enhancing ER-mitochondria signaling via expression of VAPB or PTPIP51 alleviates mutant TDP43 induced damage to IP3 receptor delivery of Ca^2+^ to mitochondria and associated synaptic defects. We also show that UDCA, an FDA approved drug linked to FTD/ALS therapy but whose mechanism of action is unclear [19, 59], stimulates VAPB-PTPIP51 binding and corrects TDP43 linked damage to the VAPB-PTPIP51 interaction and IP3 receptor delivery of Ca^2+^ to mitochondria. Finally, we show that UDCA inhibits GSK3β which is a known mediator of TDP43 toxicity and which regulates VAPB-PTPIP51 binding [41, 46, 47].

## Materials and methods

### Plasmids and lentivirus

Myc-tagged human VAPB, HA-tagged human PTPIP51 and control plasmid containing *Escherichia coli* chloramphenicol acetyltransferase in pCI-neo, and EGFP-tagged TDP43-Q331K and TDP43-A382T in pEGFP-C1 were all as described previously [47]. VAPB and PTPIP51 NanoBit plasmids were generated by cloning VAPB into pBiT1.1-N[TK/LgBiT] and pBiT2.1-N[TK/SmBiT], and PTPIP51 into pBiT1.1-C[TK/LgBiT] and pBiT2.1-C[TK/SmBiT] vectors (Promega) so as to create N-terminal fused Large/Small-BiT VAPB and C-terminal fused Large/Small-BiT PTPIP51 plasmids. Bicistronic vectors containing Myc-tagged TDP43-IRES-EGFP and HA-tagged VAPB and PTPIP51 were synthesised by GenScript. For creation of lentivirus, TDP43-IRES-EGFP, VAPB-HA and HA-PTPIP51 fragments were amplified using Q5 High-Fidelity DNA Polymerase (New England Biolabs) and cloned into lentiviral backbone vector pRRLSIN.cPPT.PGK-GFP.WPRE using NEBuilder HiFi DNA Assembly Master Mix (New England Biolabs). Primers used for amplifications were: 5’-tctagaggatccaccggtcgGTCGACCCACCATGGAGC-3’ and 5’-tgattgtcgacgcggccgctGCGGCCGCTTACTTGTAC-3’ for TDP43-IRES-EGFP; 5’-tctagaggatccaccggtcgCCACCATGTACCCATACG-3’ and 5’-tgattgtcgacgcggccgctCTACAAGGCAATCTTCCC-3’ for VAPB-HA; 5’-tctagaggatccaccggtcgCCACCATGTCTAGACTGG-3’ and 5’-tgattgtcgacgcggccgctTTAAGCGTAATCTGGAACATC-3’ for HA-PTPIP51. For TDP43 vectors, Q331K and A382T mutations were then introduced using a Q5 Site-Directed Mutagenesis Kit (New England Biolabs) using primers 5’-GGCAGCACTAAAGAGCAGTTGG-3’ and 5’-TGGGCGGCAGCCATCATG-3’ (TDP43-Q331K) and 5’-TTCTGGTGCAACAATTGGTTG-3’ and 5’-TTAGAGCCACTATAAGAGTTATTTC-3’ (TDP43-A382T). Lentiviruses were prepared as described and viral titer determined using a qPCR Lentivirus Titer Kit (ABM) [2].

### Antibodies and other reagents

Rabbit and rat antibodies to VAPB and PTPIP51 were as described and used at 1:200 for PLAs [10]. Mouse anti-VDAC1 was from Abcam (ab14734, RRID:AB 443,084) and rabbit anti-IP3 receptor type-1 was from Synaptic Systems (117,003, RRID:AB 2,619,787) and were used for PLAs at 1:200 and 1:100 respectively. Chicken anti-β-Tubulin Isotype III antibody was from Millipore (AB9354, RRID:AB 570,918) and used at 1:500 for immunostaining. Mouse anti-HA epitope-tag (6F2) and mouse anti-Myc epitope tag (9B11) antibodies were from Cell Signaling (H9658, RRID:AB 260,092; 2276 RRID:AB 331,783) and used at 1:500 for immunostaining and 1:1000 for immunoblots. Chicken anti-microtubule-associated protein-2 (MAP2) was from Genetex (GTX82661, RRID:AB 11,172,558) and used for immunostaining at 1:500. Rabbit anti-EGFP was from Invitrogen (A-11,122, RRID:AB 221,569) and used at 1:500. Rabbit anti-serine-9 phosphorylated GSK3β was from Cell Signaling (5558, RRID:AB 10,013,750) and used at 1:100. Species specific goat and donkey anti-mouse, anti-rabbit and anti-chicken Igs coupled to AlexaFluor-488, − 555, -594 or -647 were from Invitrogen and Jackson ImmunoResearch Labs.

### Cell culture, transfection and viral transduction

SH-SY5Y cells and human embryonic kidney-293 (HEK293) cells were obtained from European Collection of Cell Cultures. HEK293 cells were grown in Dulbecco’s modified Eagle’s medium with 4.5 g/l glucose and SH-SY5Y cells were grown in Dulbecco’s modified Eagle’s medium/F12 (1:1) containing 3.15 g/l glucose (Gibco). Both media were supplemented with 10% (v/v) foetal bovine serum, Glutamax, 100 IU/ml penicillin, and 100 µg/ml streptomycin (Gibco). Cells were transfected with plasmids using Lipofectamine 2000 (ratio 1 µg DNA: 2 µl Lipofectamine 2000) according to the manufacturer’s instructions (Invitrogen). SH-SY5Y cells were analysed 36 h post-transfection. Primary rat cortical neurons were obtained from embryonic day 18 rat embryos, plated on poly-D-lysine coated glass coverslips (Marienfeld) and cultured in neurobasal medium containing B27 supplement, GlutaMAX, 100 IU/ml penicillin and 100 µg/ml streptomycin (Gibco). For FM 4–64 release assays, neurons were transfected with Lipofectamine 2000 as described above at DIV7 or 8 and analysed 24 h post transfection. This timing is line with other studies of synaptic vesicle release using FM4-64 in cortical and hippocampal neurons including those that analysed FTD/ALS linked genes [26, 44]. For dendritic spine analysis, neurons were transduced with lentiviral vectors on DIV12 and analysed on DIV15; dendritic spines display a mature phenotype at this age [17]. For UDCA treatment, neurons were transfected at DIV7 and treated 24 h later with UDCA (obtained from Sigma and dissolved in DMSO) or DMSO vehicle for 24 h.

In line with previous studies, we selected cells for analyses that express relatively low levels of transfected proteins (as judged by fluorescent signal) so as to avoid any possible artefacts produced by high levels of expression [1, 34, 36, 55–53].

### FM 4–64 synaptic vesicle release assays and analyses of dendritic spines

FM 4–64 release assays were performed essentially as described [13, 17, 25]. Briefly, 24 h post transfection, neurons were washed in external solution comprising 145 mM NaCl, 2 mM KCl, 5 mM NaHCO_3_, 1 mM MgCl_2_, 2.5 mM CaCl_2_, 10 mM glucose in 10 mM HEPES buffer pH 7.25 and then incubated in external solution containing 5 µM FM 4–64 (Invitrogen), 50 µM DL-2-amino-5-phosphonovaleric acid (D-AP5) and 10 mM 6-cyano-7-nitroquinoxaline-2,3-dione (CNQX) (Tocris) for 2 min. FM 4–64 loading was stimulated by replacement of external solution with high K^+^ load external solution (97 mM NaCl, 50 mM KCl, 5 mM NaHCO_3_, 1 mM MgCl_2_, 2.5 mM CaCl_2_, 10 mM glucose, 50 mM AP5, 10 mM CDQX in 10 mM HEPES, pH 7.25) containing 5 µM FM 4–64 for 2 min. Cells were subsequently incubated for 10 min in 5 µM FM 4–64 dye in external solution to regain baseline followed by washing in external solution to remove excess dye. Synaptic vesicle FM 4–64 release was stimulated by incubation with high K^+^ release external solution containing 31.5 mM NaCl, 90 mM KCl, 5 mM NaHCO_3_, 1 mM MgCl_2_, 2.5 mM CaCl_2_, 10 mM glucose, 50 µM AP5, 10 µM CDQX in 10 mM HEPES, pH 7.25). FM 4–64 dye release was monitored by time-lapse as described [17] using a Nikon Eclipse Ti-2 microscope equipped with an Intenslight C-HGFI light source, CFI Apo Lambda S 60x/1.40 objective, TiND6 PFS-S Perfect Focus Unit and an Andor TuCam camera adapter system with EGFP/DsRed dual filter sets (Chroma Technology) and two Andor Neo sCMOD cameras; temperature was maintained at 37 °C using a microscope incubation chamber (Solent Scientific). Recordings were obtained at 5 s time-lapse intervals using Nikon NIS-Elements AR software and analyses performed using ImageJ. Synaptic vesicle release was expressed as fluorescence signals after KCl treatment relative to the average baseline prior to treatment (F/F0).

Dendritic spine densities were quantified as described previously [17]. Briefly, cells were fixed in 4% paraformaldehyde and following immunostaining, z-plane images with 0.2 μm intervals were captured using a Nikon Eclipse Ti-E Inverted microscope with 100 × 1.49 NA CFI objective and an Andor iXon EMCCD camera equipped with Visitech iSIM Super Resolution System as described [17, 18, 37]. Spine densities were quantified using Neuronlucida TM software (MBF Bioscence, VT USA).

### SDS-PAGE and immunoblotting

Cells were prepared for SDS-PAGE and analysed by immunoblotting essentially as described previously [35]. Briefly, cells were washed with phosphate buffered saline (PBS) and harvested by scraping into ice-cold radioimmunoprecipitation (RIPA) lysis buffer containing 150 mM NaCl, 1% Triton X-100, 0.5% sodium deoxycholate, 0.1% SDS in 50 mM Tris-HCl pH 8 with protease inhibitors (Complete, Roche). SDS-PAGE sample buffer was then added and samples heated at 96 ºC for 5 min. Samples were separated on 10% gels using a Mini-PROTEAN 2 gel electrophoresis systems (Bio-Rad) with a discontinuous buffer system. Separated proteins were transferred to BioTrace NT nitrocellulose membrane (0.2 μm pore size; Pall Corporation) using a Mini Trans-Blot electrophoretic transfer cell (Bio-Rad) for 1.5 h. Membranes were blocked with Tris-HCl buffered saline (TBS) containing 5% (w/v) milk powder and 0.1% (v/v) Tween-20 for 1 h. Membranes were probed with primary antibodies in blocking buffers supplemented with 0.1% (v/v) Tween-20 (TBS/Tween-20), washed in TBS/Tween-20 and incubated with IRDye-conjugated secondary antibodies in wash buffer and proteins visualised using an Odyssey CLx near infrared imaging system (Li-Cor Biosciences).

### Calcium measurements

Mitochondrial Ca^2+^ levels were measured with Rhod-2 AM (Invitrogen) essentially as described previously [10, 16, 37, 40, 47, 48]. Briefly, SH-SY5Y cells were plated on coverslips (Marienfeld) and 24 h post transfection, selected for analyses via EGFP signal. Cells were loaded with 2 µM Rhod-2 AM in external solution (145 mM NaCl, 2 mM KCl, 5 mM NaHCO_3_, 1 mM MgCl_2_, 2.5 mM CaCl_2_, 10 mM glucose, 10 mM Na-HEPES pH 7.25) in the presence of 0.02% (v/v) Pluronic-F27 (Invitrogen) for 15 min. After removing dye, cells were washed with external solution for 15 min at 37 °C, mounted in a Ludin imaging chamber (Life Imaging Systems, Basel, Switzerland) and kept under constant perfusion in external solution (0.5 ml/min) using an Ismatec REGLO peristaltic pump (IDEX Corporation, Glattbrugg, Switzerland). To invoke Ca^2+^ efflux from ER stores and measure changes in Ca^2+^ levels, 100 µM oxotremorine-M (Tocris) was applied in external solution. Rhod-2 AM images were recorded in time-lapse mode (2 s interval, 100 ms exposure) using a Nikon Eclipse Ti-2 microscope driven by NIS Elements AR software and equipped with Intenslight C-HGFI light source, CFI Plan Fluor 40x/1.4 NA objective, Andor Neo scientific complementary metal-oxide-semiconductor camera (Andor Technology). Filter sets were from Chroma Technology. Data were analysed with NIS Elements AR software. Mitochondrial and Ca^2+^ levels were expressed as fluorescence signals after oxotremorine-M treatment relative to average baseline before oxotremorine-M application (F/F0).

### Proximity ligation assays and immunostaining

Proximity Ligation Assays (PLAs) to detect IP3R1-VDAC1 or VAPB-PTPIP51 interactions were performed as described previously using Duolink In Situ Orange Kits (Sigma) according to manufacturer’s instructions [17, 18, 35]. After amplification steps, samples were immunostained with β-tubulin III (SHSY5Y cells) or MAP2 (neurons) to confirm neural identity.

To detect serine-9 phosphorylated GSK3β, cells were fixed in 4% paraformaldehyde, washed three times in Tris-HCl buffered saline (TBS), permeabilised with 0.1% Triton X-100 in TBS for 15 min and blocked in 5% bovine serum albumin (BSA) in TBS for 1 h. Cells were then labelled with primary antibody diluted in 1% BSA in TBS overnight at 4 °C, washed three times with 0.01% Triton X-100/TBS, incubated with secondary antibodies diluted in TBS, washed three times with TBS and mounted in aqueous mounting medium with DAPI (Abcam). Z-plane images with 0.3 μm intervals were captured using a Nikon Eclipse Ti-E Inverted microscope with CFI Apo Lambda S 60x/1.40 objective and an Andor iXon EMCCD camera equipped with Visitech iSIM Super Resolution System. Mean cytoplasmic fluorescent intensities were quantified using ImageJ.

### NanoBiT assays

NanoBiT assays were performed on SH-SY5Y cells cultured in white opaque 96-well plates at initial density of 30,000 cells/well. Assays were performed in living cells according to the manufacturerµ’s instruction (Promega). Briefly, cells were transfected with the 100 ng NanoBiT plasmids as described above and 24 h later, treated with vehicle (DMSO) or UDCA for 24 h. Medium was replaced with 100 µl OptiMEM medium without phenol red (Gibco) and NanoBiT luminescence signals obtained by addition of 25 µl reconstituted NanoGlo Live Assay substrate to each well and quantified using a GloMax Navigator luminometer for 15 min (0.5s integration time/well).

### GSK3β ELISAs

ELISAs for quantifying total and serine-9 phosphorylated GSK3β (CST7265C PathScan® Total GSK3β Sandwich ELISA; CST7311C PathScan® Phospho-GSK3β (Ser9) sandwich ELISA) were obtained from Cell Signaling Technology. UDCA and vehicle (DMSO) treated neurons were processed according to the manufacturer’s instructions. Briefly, neurons were washed with PBS and lysed in kit cell lysis buffer (CST9803) containing 1 mM phenylmethylsulfonyl fluoride, protease and phosphatase inhibitors (Complete, Roche). Protein concentrations were determined using Pierce™ BCA Protein Assay Kit (Thermo Fisher Scientific) and an equal amount of total protein (0.15 mg/ml for phospho-GSK3β and 0.045 mg/ml for total GSK3β ELISAs) in 100 µl kit supplied sample buffer loaded in duplicates to the ELISA microwells. Following a 2 h incubation at 37 °C, wells were washed in kit supplied wash buffer and 100 µl of detection antibody added and samples incubated for 1 h at 37 °C. After washing, 100 µl of reconstituted HRP-linked secondary antibody was added to the wells and incubated for 30 min at 37 °C, followed by incubation with 3,3′,5,5′-Tetramethylbenzidine substrate for 10 min at 37 °C. Upon addition of stop solution absorbance was read at 450 nm using a CLARIOstar multiplate reader (BMG LabTech).

### Statistical analyses

Statistical analysis was performed using Excel (Microsoft Corporation) and Prism software (version 9.3.1; GraphPad Software Inc.). Details are described in the Figure legends.

## Results

### Expression of VAPB or PTPIP51 rescues mutant TDP43-induced disruption to ER-mitochondria Ca^2+^ delivery

A primary function of the VAPB-PTPIP51 tethers is to facilitate delivery of Ca^2+^ from ER stores to mitochondria; this delivery regulates mitochondrial ATP production and downstream bioenergetic linked functions including synaptic activity [10, 18–17, 37, 40, 47, 48]. ER-mitochondria Ca^2+^ delivery involves its release from ER located IP3 receptors and its uptake via the mitochondrial voltage-dependent anion channel-1 (VDAC1) [6, 32, 33, 39]. Thus, siRNA loss of VAPB or PTPIP51 reduces ER-mitochondria tethering and so inhibits mitochondrial Ca^2+^ delivery whereas overexpression of VAPB or PTPIP51 increases tethering to enhance Ca^2+^ delivery [10, 16, 37, 47].

As detailed above, both wild-type and FTD/ALS mutants of TDP43 disrupt the VAPB-PTPIP51 interaction, ER-mitochondria contacts, IP3 receptor delivery of Ca^2+^ to mitochondria and activate GSK3β to similar extents, and induce indistinguishable disease phenotypes in transgenic mice [47, 52]. We therefore studied how VAPB/PTPIP51 expression affects damage to ER-mitochondria Ca^2+^ delivery induced by two familial FTD/ALS mutants, TDP43-Q331K or TDP43-A382T; both of these mutants have been shown to damage the VAPB-PTPIP51 interaction, ER-mitochondria contacts and IP3 receptor delivery of Ca^2+^ to mitochondria [47].

**Fig. 1:**
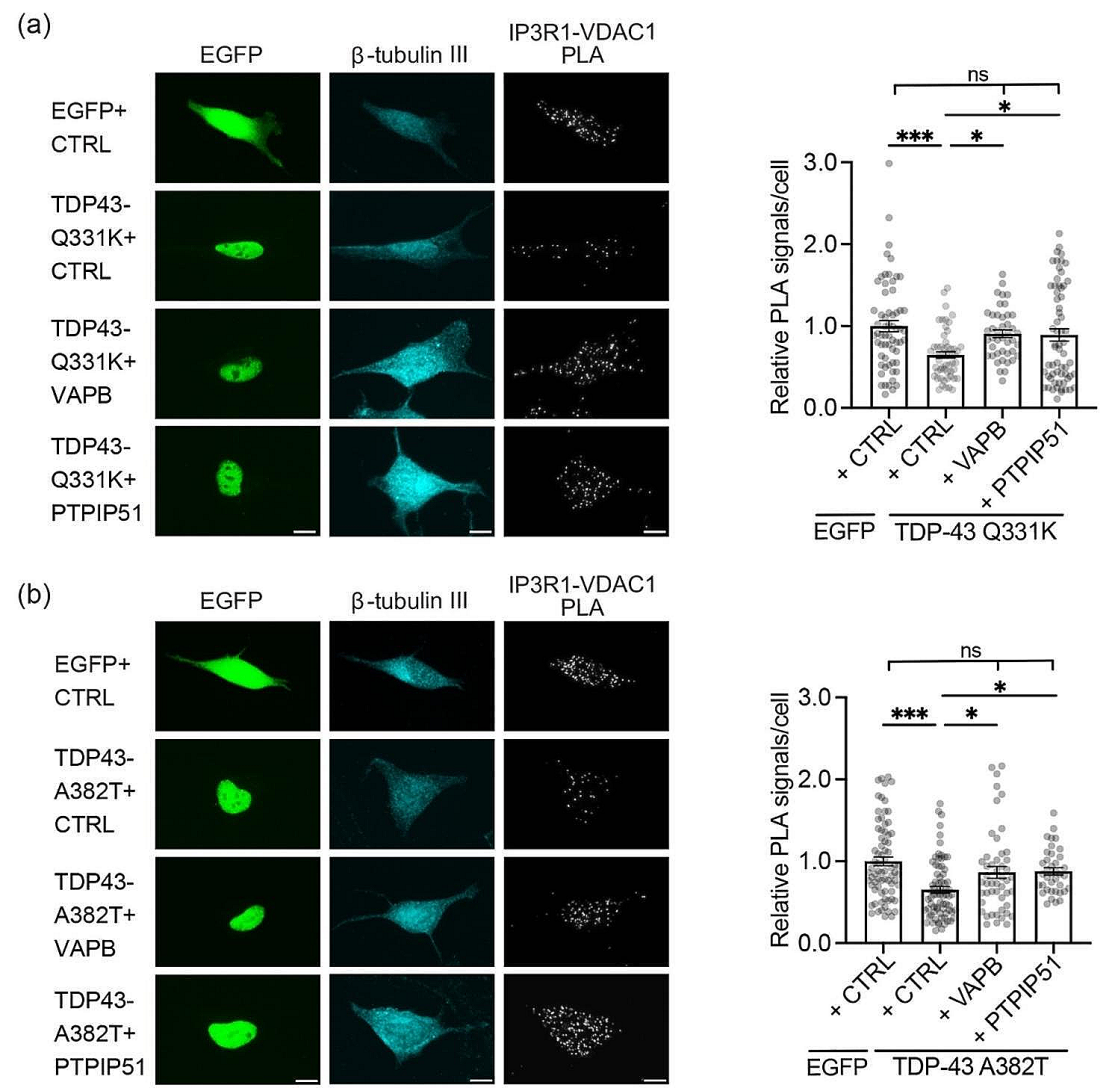
Expression of VAPB or PTPIP51 rescues mutant TDP43 induced disruption to the IP3 receptor-VDAC1 interaction. (**a** and **b**) Representative images of IP3 receptor type1-VDAC1 PLAs in SH-SY5Y cells transfected with EGFP + control (CTRL) vector, EGFP-tagged-TDP43-Q331K or -TDP43-A382T + control vector and EGFP-tagged TDP43-Q331K/EGFP-TDP43-A382T + either VAPB or PTPIP51 as indicated. TDP43 was identified via the EGFP tag and cells immunostained for β-tubulin III (artificially shown in cyan). Scale bars = 10 μm. Bar charts show PLA signals per cell after normalisation to control EGFP + CTRL cell data. Data were analysed by one-way ANOVA with Tukey’s post hoc test; (**a**) *N* = 43–63 cells from 3 independent experiments, (**b**) *N* = 38–75 cells from 3 independent experiments. Error bars are SEM; * *p*≤0.05, *** *p*≤0.001, ns not significant

For these experiments, we utilised neuronal SH-SY5Y cells. These cells have been used in many previous studies that investigated the roles of the VAPB-PTPIP51 tethers in IP3 receptor Ca^2+^ delivery to mitochondria e.g. [18, 37, 40, 49]. Use of SH-SY5Y cells therefore permits accurate comparisons with previous studies. In the first instance, we used proximity ligation assays (PLAs) to monitor whether expression of VAPB or PTPIP51 can rescue mutant TDP43 induced disruption to the IP3 receptor-VDAC1 channel; PLAs have been used in numerous studies to quantify changes in IP3 receptor-VDAC1 interactions e.g. [3, 16, 18, 22]. As predicted from previous work [47], expression of familial FTD/ALS mutant TDP43-Q331K or TDP43-A382T significantly reduced IP3 receptor-VDAC1 PLA signals in these experiments. However, co-expression of either VAPB or PTPIP51 rescued these defects (Fig. 1a, b).

**Fig. 2:**
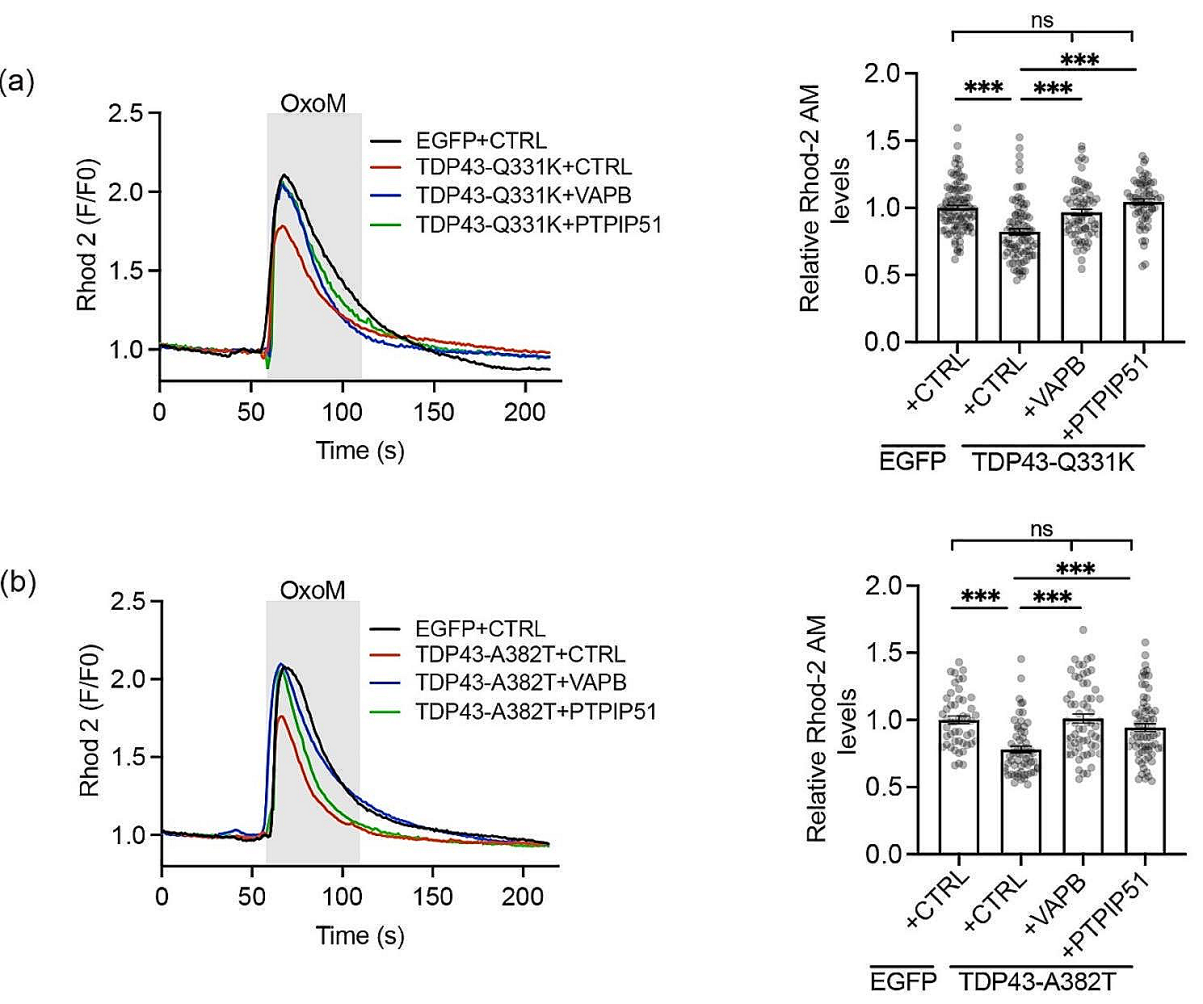
Expression of VAPB or PTPIP51 rescues mutant TDP43 induced disruption to IP3 receptor mediated delivery of Ca^2+^ to mitochondria. (**a** and **b**) SH-SY5Y cells were transfected with EGFP + control (CTRL) vector, EGFP-TDP43-Q331K or TDP43-A382T + control vector and EGFP-TDP43-Q331K/EGFP-TDP43-A382T + either VAPB or PTPIP51 as indicated. Cells were loaded with Rhod-2 AM and Ca^2+^ release induced by treatment with oxotremorine-M (OxoM). Representative Rhod-2 AM fluorescence traces are shown on the left with OxoM treatment depicted by shaded area; normalised peak values are shown in the bar charts on the right. Expression of TDP43-Q331K or TDP43-A382T reduced mitochondrial Ca^2+^ levels and this was rescued by VAPB/PTPIP51 expression. Data were analysed by one-way ANOVA with Tukey’s post hoc test. *N* = 62–96 cells from 3 independent experiments in (**a**) and *N* = 48–69 cells from 3 independent experiments in (**b**). Error bars are SEM; *** *p*≤0.001, ns not significant

We next enquired whether VAPB and/or PTPIP51 expression rescued mutant TDP43 damage to IP3 receptor linked Ca^2+^ delivery to mitochondria. We stimulated IP3 receptor Ca^2+^ release by treatment of cells with the muscarinic acetylcholine receptor agonist oxotremorine-M and monitored mitochondrial Ca^2+^ uptake using the indicator dye Rhod-2 AM. Again this approach involving oxotremorine-M stimulation in SH-SY5Y cells has been used in many previous studies on VAPB-PTPIP51 regulation of ER-mitochondria Ca^2+^ exchange and so permits proper comparison with previous work [10, 16, 18, 37, 40, 47, 48]. In line with these studies, oxotremorine-M induced a time-dependent increase in mitochondrial Ca^2+^ levels but compared to control cells, the peak levels were significantly lower in cells expressing TDP43-Q331K or TDP43-A382T [47] (Fig. 2a, b). Notably however, co-expression of either VAPB or PTPIP51 corrected these defects (Fig. 2a, b). Thus, expression of VAPB or PTPIP51 to enhance ER-mitochondria tethering and signaling, rectifies mutant TDP43 induced damage to the IP3 receptor-VDAC1 channel, and ER-mitochondria Ca^2+^ delivery.

### Expression of VAPB or PTPIP51 rescues mutant TDP43-induced synaptic damage

Like all major neurodegenerative diseases, damage to synaptic function is a key feature of FTD/ALS and this includes both pre- and post-synaptic defects [15, 23]. Mutant TDP43 perturbs synaptic vesicle release and induces loss of dendritic spines [4, 12]. We therefore monitored how expression of VAPB or PTPIP51 affected this damage in cultured rat cortical neurons; cortical neurons are affected in FTD.

**Fig. 3:**
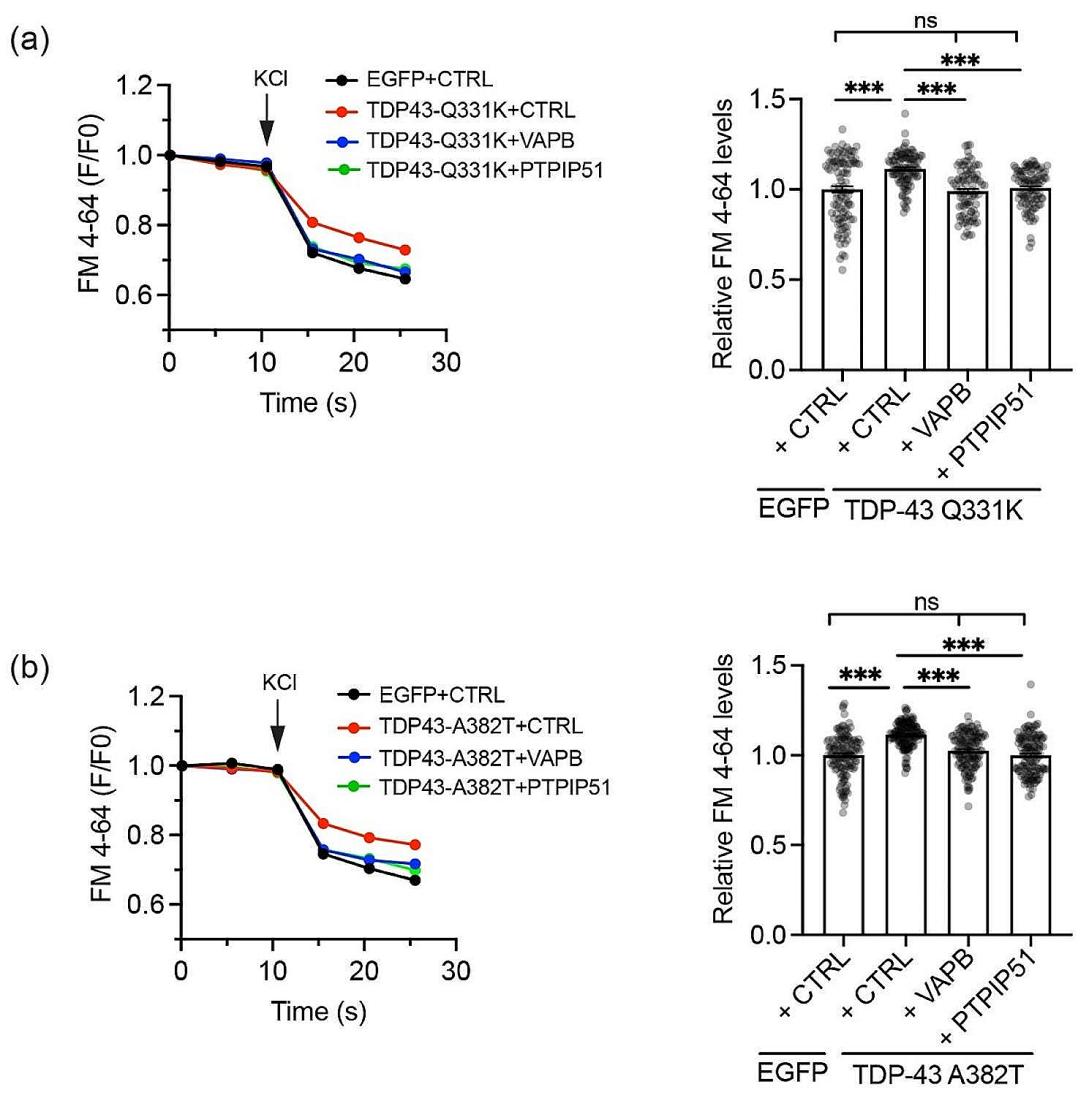
Expression of VAPB or PTPIP51 rescues mutant TDP43 induced disruption to synaptic vesicle release. (**a** and **b**) Kinetics of FM 4–64 release from synaptic boutons in cortical neurons transfected with (**a**) EGFP + CTRL, EGFP + TDP43-Q331K + CTRL or EGFP + TDP43-Q331K + either VAPB or PTPIP51 and (**b**) EGFP + CTRL, EGFP + TDP43-A382T + CTRL or EGFP + TDP43-A382T + either VAPB or PTPIP51 as indicated. Synaptic vesicle release was induced by treatment with KCl and FM 4–64 signals were determined from images acquired by time-lapse microscopy. Representative FM 4–64 traces are shown on the left; bar charts show F/F0 FM 4–64 fluorescent signals 5 s post KCl treatment. Data were analysed by one-way ANOVA with Tukey’s post hoc test. *N* = 68–102 boutons in (**a**) and *N* = 133–146 boutons in (**b**) from 3 independent experiments. Error bars are SEM; *** *p*≤0.001, ns not significant

To study synaptic vesicle release, we utilised the red fluorescent synaptic vesicle recycling dye FM 4–64. This is taken into synaptic vesicles as they form via endocytosis but induction of synaptic vesicle release causes a quantifiable loss of FM 4–64 fluorescent signal [13]. Such FM 4–64 assays have been used previously to monitor the effects of VAPB and PTPIP51 on synaptic vesicle release [17]. For these experiments, we transfected neurons with EGFP + control vector, EGFP + either TDP43-Q331K or TDP43-A382T + control vector, or EGFP + TDP43-Q331K/TDP43-A382T + either VAPB or PTPIP51. Co-transfection with EGFP is required to discern cell shape so as to permit proper identification of synaptic boutons for analyses. All neurons received the same amount of plasmid. We induced synaptic vesicle release by treatment with KCl and quantified FM 4–64 signals by time-lapse microscopy as described previously [17]. As predicted from previous studies [4], compared to controls, TDP43-Q331K and TDP43-A382T both markedly inhibited FM 4–64 release (Fig. 3a, b). However, co-expression of either VAPB or PTPIP51 rescued these defects (Fig. 3a, b).

**Fig. 4:**
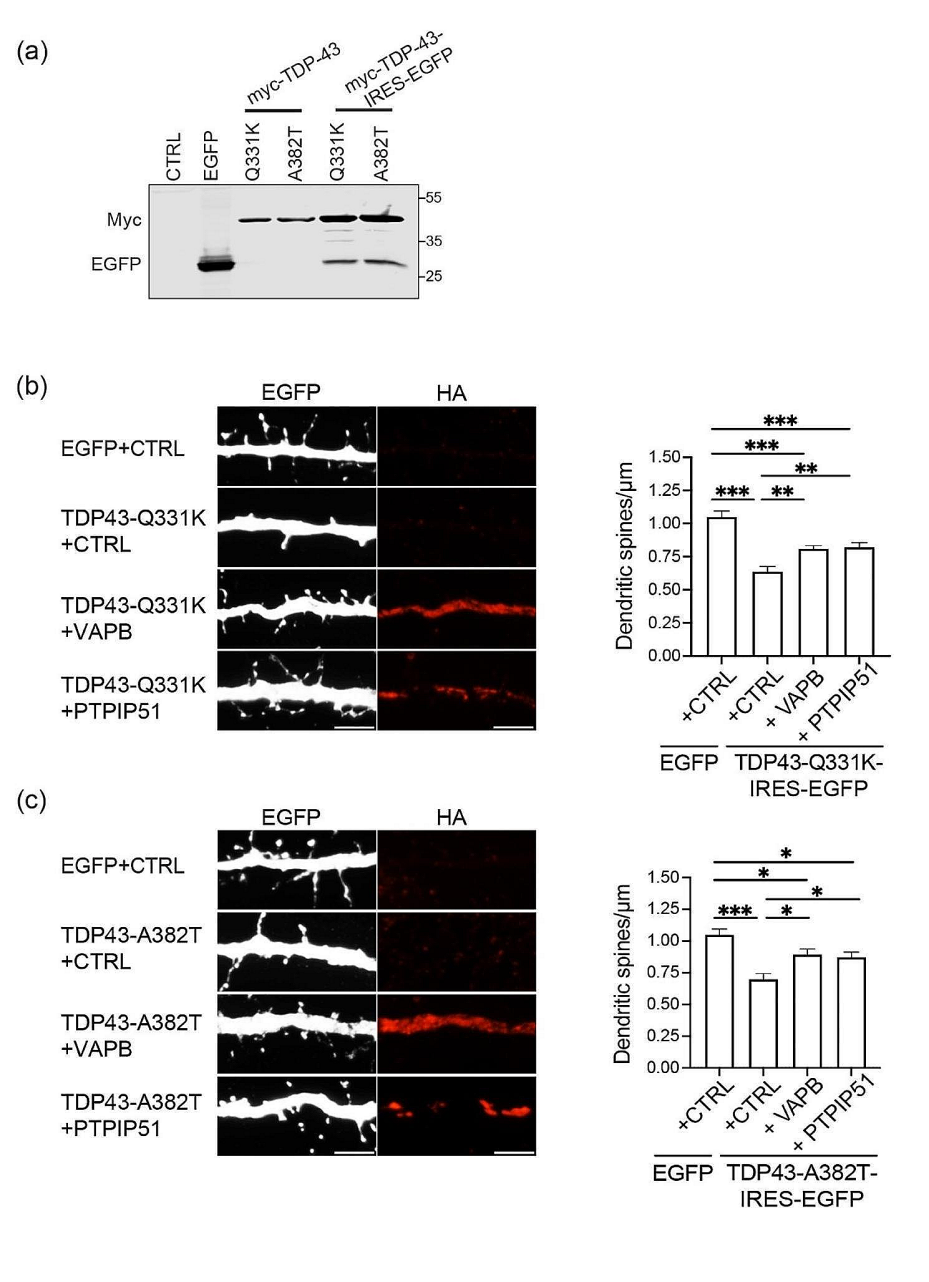
Expression of VAPB or PTPIP51 rescues mutant TDP43 induced disruption to dendritic spine numbers. (**a**) Characterisation of mutant TDP43-IRES-EGFP lentivirus. HEK293 cells were transfected with control vector (CTRL), EGFP, Myc-TDP43-Q331K or Myc-TDP43-A382T as negative and positive controls, and samples run on SDS-PAGE alongside samples of neurons infected with lentivirus expressing Myc-tagged TDP43-Q331K-IRES-EGFP or TDP43-A382T-IRES-EGFP as indicated. Samples were probed on immunoblots for TDP43 via the Myc tags and EGFP; molecular masses are shown on the right. (**b** and **c**) Representative super resolution SIM images of dendritic spines in neurons treated with lentivirus expressing (**b**) EGFP + CTRL, TDP43-Q331K-IRES-EGFP + CTRL, TDP43-Q331K-IRES-EGFP + HA tagged VAPB or TDP43-Q331K-IRES-EGFP + HA tagged PTPIP51 or (**c**) EGFP + CTRL, TDP43-A383T-IRES-EGFP + CTRL, TDP43-A382T-IRES-EGFP + HA tagged VAPB or TDP43-A382T-IRES EGFP + HA tagged PTPIP51. Neurons were infected with lentivirus at DIV 12 and analysed at DIV 15. Exogenous VAPB and PTPIP51 were identified by immunostaining for the HA tags. Scale bars = 5 μm. Bar charts show spine densities calculated as described (spines/µm) [17]. Data were analysed by one-way ANOVA with Tukey’s post hoc test. *N* = 20–26 neurons from 3 independent experiments; error bars are SEM. * *p*≤0.05, ** *p*≤0.01, *** *p*≤0.001

We next monitored how expression of VAPB or PTPIP51 affected dendritic spine loss induced by mutant TDP43 in the cortical neurons. For these experiments we generated lentivirus to express EGFP (to visualise neuronal architecture including dendritic spines) + either control or Myc-tagged TDP43-Q331K or TDP43-A382T. Co-expression of mutant TDP43 and EGFP was achieved by use of an internal ribosome entry site (IRES); immunoblots for EGFP and Myc-TDP43 confirmed expression of both proteins (Fig. 4a). We also generated lentivirus expressing hemagglutinin (HA) tagged VAPB or PTPIP51 (HA-VAPB, PTPIP51-HA). Neurons were infected with EGFP + control, TDP43-Q331K- or TDP43-A382T-IRES-EGFP viruses with either control, HA-VAPB or PTPIP51-HA virus. Neurons expressing TDP43 were identified by EGFP signal and HA-VAPB or PTPIP51-HA expression confirmed by immunostaining for the HA tags. As predicted, expression of TDP43-Q331K or TDP43-A382T alone induced a marked decrease in dendritic spine numbers. However, co-expression of HA-VAPB or PTPIP51-HA significantly reduced this decrease (Fig. 4b, c). Thus, expression of VAPB or PTPIP51 to stimulate ER-mitochondria signaling, corrects TDP43 induced damage to synaptic vesicle release and at least partially corrects TDP43 damage to dendritic spine loss.

### UDCA rescues mutant TDP43-induced disruption to the VAPB-PTPIP51 interaction

**Fig. 5:**
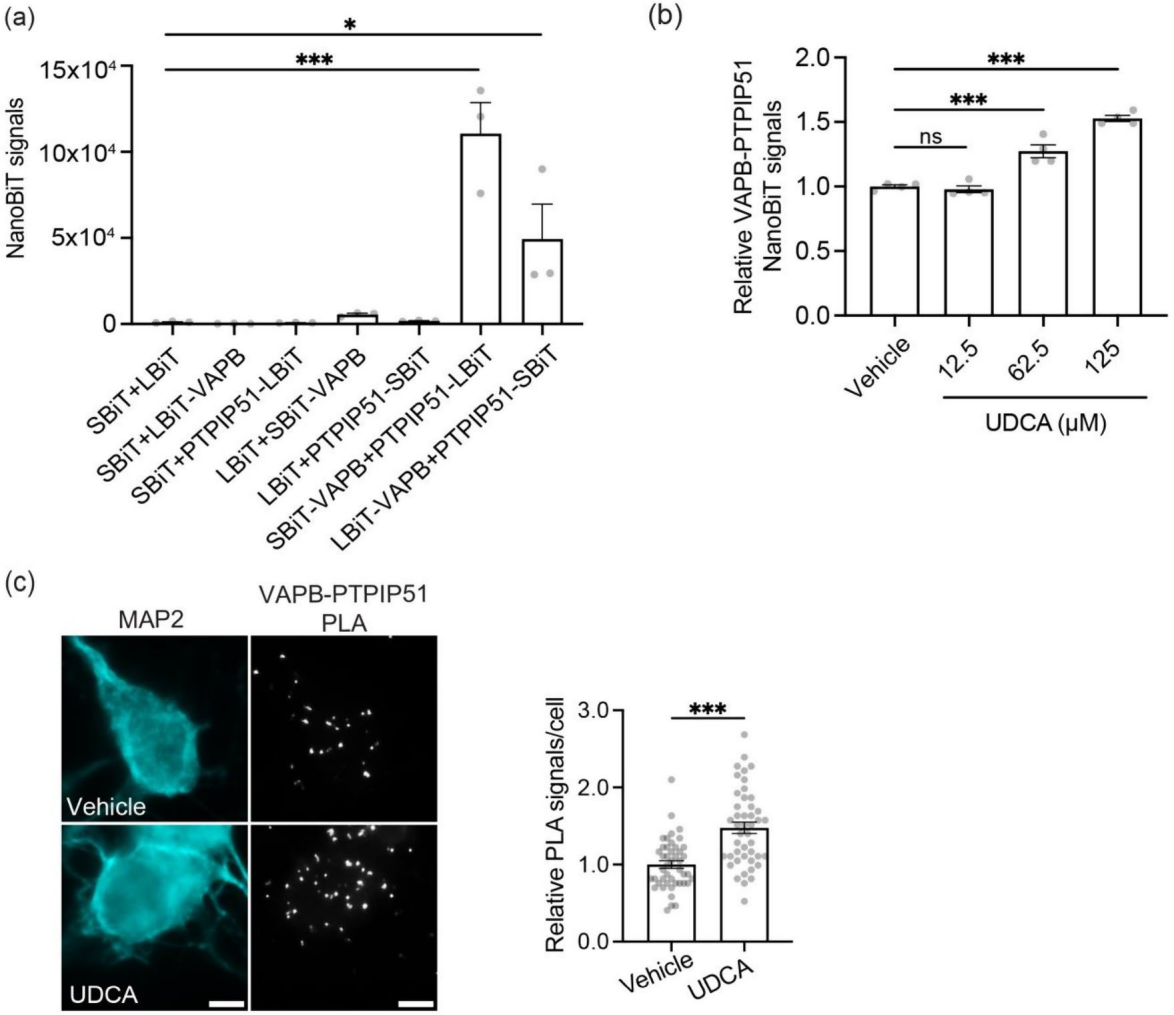
UDCA stimulates the VAPB-PTPIP51 interaction. (**a**) Characterisation of VAPB-PTPIP51 NanoBiT assays. SH-SY5Y cells were transfected with either empty Small and Large BiT plasmids (SBiT + LBiT), SBiT + LBiT-VAPB, SBiT + LBiT-PTPIP51, LBiT + SBiT-VAPB, LBiT + PTPIP51-SBiT, SBiT-VAPB + PTPIP51-LBiT or LBiT-VAPB + PTPIP51-SBiT as indicated. Signals above background were only detected in cells transfected with SBiT-VAPB + PTPIP51-LBiT or LBiT-VAPB + PTPIP51-SBiT and the highest signals were detected in SBiT-VAPB + PTPIP51-LBiT cells. Data were analysed by one-way ANOVA with Tukey’s post hoc test. Data are from 3 independent experiments each with *N* = 6; error bars are SEM. * *p*≤0.05, *** *p*≤0.001 (**b**) UDCA stimulates VAPB-PTPIP51 binding in NanoBiT assays. SH-SY5Y cells were transfected with SBiT-VAPB + PTPIP51-LBiT and then treated with vehicle or increasing concentrations of UDCA for 24 h. 62.5 µM and 125 µM UDCA both stimulated VAPB-PTPIP51 binding. Data were analysed by one-way ANOVA with Tukey’s post hoc test. Data are from 4 independent experiments each with *N* = 6. Error bars are SEM. *** *p*≤0.001, ns not significant. (**c**) UDCA stimulates VAPB-PTPIP51 binding in cortical neurons. Representative images of VAPB-PTPIP51 PLA signals in neurons treated with vehicle or 62.5 µM UDCA for 24 h. Cells were immunostained for MAP2 (artificially shown in cyan) to confirm neuronal identity Scale bars = 5 μm. Data were analysed by unpaired t-test. *N* = 45 cells from 3 independent experiments. Error bars are SEM, *** *p*≤0.001, ns not significant

UDCA is an FDA approved drug that is licenced for the treatment of primary biliary cholangitis. However, there is increasing interest in its neuroprotective effects in neurodegenerative diseases and clinical trials are underway with UDCA and related compounds for Alzheimer’s disease, Parkinson’s disease and ALS [19, 59]. Despite this interest, its precise mechanism of action is unclear and it has been linked to protection against mitochondrial damage, ER stress, apoptosis, autophagy and inflammation [19, 38, 59]. Since these functions are all regulated by ER-mitochondria signaling [6, 32, 39], we speculated that UDCA might influence the VAPB-PTPIP51 interaction. To begin to test this possibility, we first used Nanoluc Binary Technology (NanoBiT) luciferase complementation assays [9]. This involved fusion of Large and Small BiT fragments of Nanoluciferase to the N- and C-termini of VAPB and PTPIP51 respectively (i.e. the VAPB/PTPIP51 domains that project into the cytoplasm), and quantification of luciferase signals following transfection into SH-SY5Y cells. Binding of VAPB to PTPIP51 in the transfected cells brings the NanoBiT fragments into close proximity to generate functional luciferase whose signals correlate with the strength of the interaction. We tested fusing the Large and Small BiT fragments to either VAPB or PTPIP51. In control experiments involving transfections into SH-SY5Y cells, significant luciferase signals were only generated when both VAPB and PTPIP51 NanoBiT fusion plasmids were expressed, and the highest signals were obtained with VAPB fused to Small and PTPIP51 to Large BiTs; this combination was used in all future experiments (Fig. 5a). We then used the assay to determine how treatment with increasing concentrations of UDCA affected VAPB-PTPIP51 binding; UDCA at 62.5 µM and 125 µM both significantly increased binding (Fig. 5b). These concentrations are in line with those used in many cellular studies and correlate with the levels of UDCA and its metabolites detected in the serum of patients treated with UDCA; see for e.g. [5, 56]. We also monitored whether UDCA affected the VAPB-PTPIP51 interaction in cultured rat cortical neurons using PLAs. Compared to vehicle, UDCA stimulated VAPB-PTPIP51 binding in these assays (Fig. 5c).

**Fig. 6:**
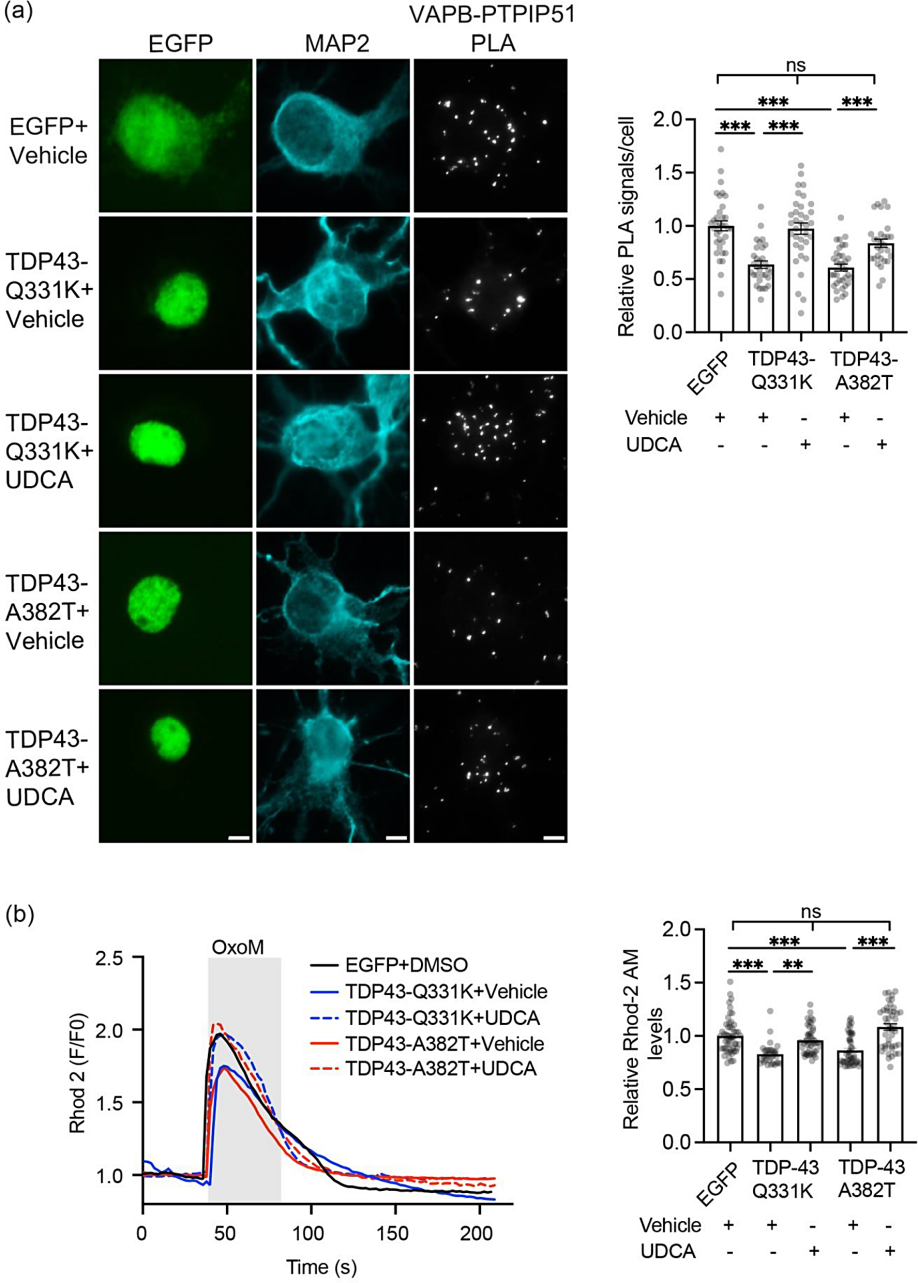
UDCA rescues mutant TDP43 induced damage to the VAPB-PTPIP1 interaction in cortical neurons and to IP3 receptor mediated delivery of Ca^2+^ to mitochondria. (**a**) Representative images of VAPB-PTPIP51 PLA signals in neurons transfected with EGFP, EGFP-TDP43-Q331K or EGFP-TDP43-A382T and treated with either vehicle or 62.5 µM UDCA for 24 h. TDP43 was identified via the EGFP tag and cells immunostained for MAP2 (artificially shown in cyan) to confirm neuronal identity. Scale bars = 5 μm. Bar charts show numbers of PLA signals per cell after normalisation to EGFP + vehicle treated control. Data were analysed by one-way ANOVA with Tukey’s post hoc test. *N* = 30–35 cells from 3 independent experiments. Error bars are SEM, *** *p*≤0.001. (**b**) UDCA rescues mutant TDP43 induced disruption IP3 receptor delivery of Ca^2+^ to mitochondria. SH-SY5Y cells were transfected with EGFP control, EGFP-TDP43-Q331K or EGFP-TDP43-A382T and treated with vehicle or 62.5 µM UDCA for 24 h as indicated, and mitochondrial Ca^2+^ levels quantified using Rhod-2 AM. Ca^2+^ release was induced by treatment of cells with oxotremorine-M (OxoM). Representative Rhod-2 AM fluorescence traces are shown on the left with OxoM treatment depicted by shaded area; normalised peak values are shown in the bar charts on the right. Expression of TDP43-Q331K or TDP43-A382T reduced mitochondrial Ca^2+^ levels and this was rescued by UDCA. Data were analysed by one-way ANOVA with Tukey’s post hoc test. *N* = 32–53 cells from 3 independent experiments. Error bars are SEM; ** *p*≤0.01, *** *p*≤0.001, ns not significant

We next tested whether UDCA might rescue mutant TDP43 induced damage to the VAPB-PTPIP51 interaction in cultured rat cortical neurons. We transfected neurons with EGFP control vector, EGFP-TDP43-Q331K or EGFP-TDP43-A382T and treated the cells with vehicle or UDCA. In line with previous studies, mutant TDP43 disrupted VAPB-PTPIP51 binding in these assays [47] but this effect was alleviated in cells treated with UDCA (Fig. 6a).

Finally, we enquired whether UDCA also rescued mutant TDP43 induced damage to IP3 receptor linked Ca^2+^ delivery to mitochondria. As described above and in previous studies, we monitored mitochondrial Ca^2+^ uptake using the indicator dye Rhod-2 AM in SH-SY5Y cells following oxotremorine-M stimulation of IP3 receptors [10, 16, 18, 37, 40, 47, 48]. In line with these earlier studies, peak mitochondrial Ca^2+^ levels were lower in cells expressing TDP43-Q331K or TDP43-A382T. However, treatment with UDCA rescued these defects (Fig. 6b).

### UDCA inhibits mutant TDP43-induced activation of GSK3β

**Fig. 7:**
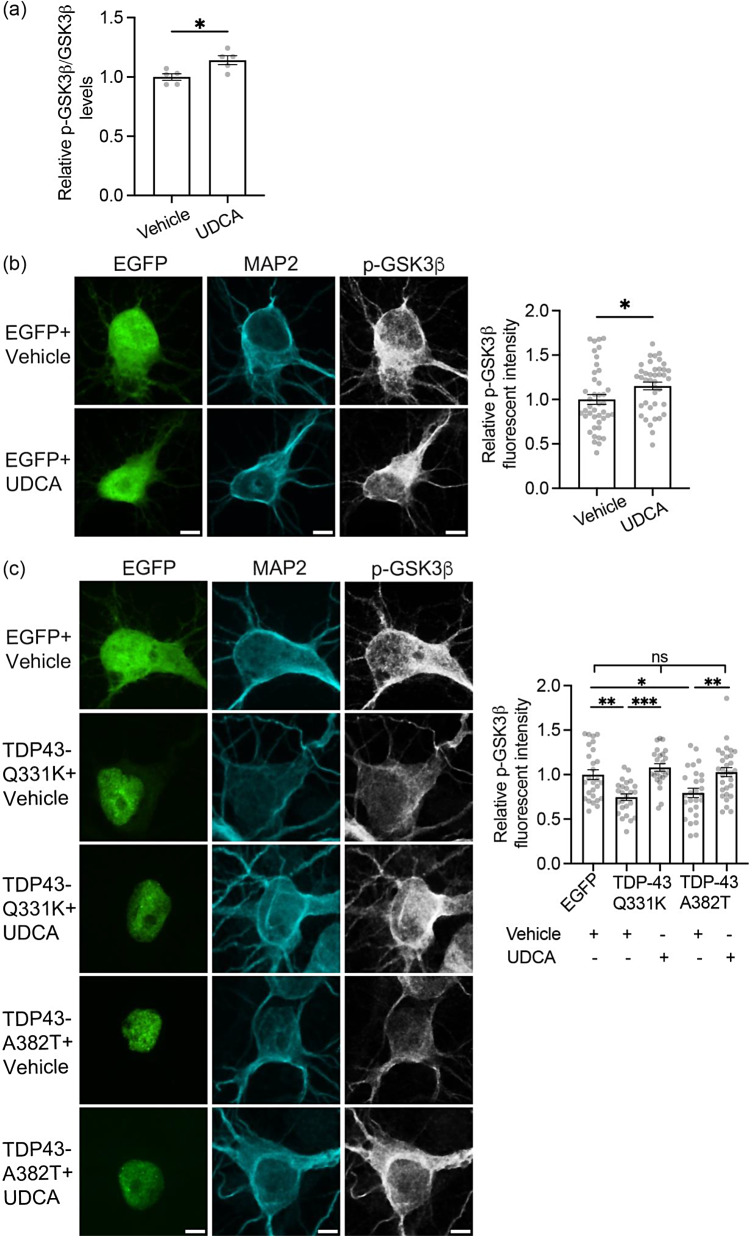
UDCA inhibits GSK3β activity and rescues mutant TDP43-induced activation of GSK3β in cortical neurons. (**a**) ELISAs to demonstrate UDCA inhibition of GSK3β. Neurons were treated with 62.5 µM UDCA for 24 h and total and serine-9 phosphorylated GSK3β (p-GSK3β) levels quantified. Bar chart shows relative serine-9 phosphorylated/total GSK3β levels. Data were analysed by unpaired t-test. *N* = 5 independent experiments. Error bars are SEM, * *p*≤0.05. (**b**) Representative images of EGFP transfected neurons immunostained for serine-9 phosphorylated GSK3β (p-GSK3β) and MAP2 (artificially shown in cyan) to confirm neuronal identity. Scale bar = 5 μm. Bar charts show mean fluorescent intensity after normalisation to EGFP + vehicle treated control. Data were analysed by unpaired t-test. *N* = 40–42 cells from 5 independent experiments. Error bars are SEM, * *p*≤0.05. (**c**) Representative images of p-GSK3b immunostained neurons transfected with EGFP control, EGFP-TDP43-Q331K or EGFP-TDP43-A382T and treated with either vehicle or 62.5 µM UDCA for 24 h. TDP43 was identified via the EGFP tag and cells immunostained for MAP2 to confirm neuronal identity. Scale bars = 5 μm. Bar charts show mean fluorescent intensity after normalisation to EGFP + vehicle treated control. Data were analysed by one-way ANOVA with Tukey’s post hoc test. *N* = 24–30 cells from 3 independent experiments. Error bars are SEM; * *p*≤0.05, ** *p*≤0.01, *** *p*≤0.001, ns not significant

A number of studies have shown that TDP43 toxicity involves, at least in part, activation of GSK3β [41, 46, 47]. A primary route for regulating GSK3β activity involves phosphorylation of serine-9; serine-9 phosphorylation inhibits GSK3β activity [27]. Moreover, disruption of VAPB-PTPIP51 binding by TDP43 involves activation of GSK3β via inhibition of serine-9 phosphorylation [47]. We therefore enquired whether UDCA might inhibit GSK3β via an effect on serine-9 phosphorylation. To do so, we first used ELISAs to quantify the relative levels of GSK3β serine-9 phosphorylation in non-tranfected cortical neurons treated with vehicle or 62.5 µM UDCA. UDCA significantly increased GSK3β serine-9 phosphorylation in these assays (Fig. 7a). We next monitored GSK3β activity in vehicle or UDCA treated neurons by quantifying GSK3β serine-9 phosphorylation immunofluorescent signals in individual neurons transfected with EGFP. Again, UDCA significantly increased GSK3β serine-9 phosphorylation in these assays (Fig. 7b). Notably, the extent of these UDCA induced increases to GSK3β serine-9 phosphorylation were highly similar in the two assays and this validates the use of the immunostaining method.

We next monitored whether UDCA treatment might inhibit TDP43 activation of GSK3β by quantifying GSK3β serine-9 phosphorylation immunofluorescent signals in mutant TDP43 transfected neurons. In line with previous studies [47], compared to control EGFP treated cells, TDP43-Q331K or TDP43-A382T both induced a significant decrease in GSK3β serine-9 phosphorylation (Fig. 7c). However, treatment with UDCA alleviated this effect (Fig. 7c). Thus, UDCA rescues mutant TDP43 induced damage to VAPB-PTPIP51 binding and IP3 receptor delivery of Ca^2+^ to mitochondria, and inhibits TDP43 linked activation of GSK3β which is a known mediator of TDP43 toxicity.

## Discussion

Communications between ER and mitochondria regulate many of the physiological processes that are damaged in FTD/ALS and studies from multiple research groups have now shown that ER-mitochondria signaling is disrupted by a number of FTD/ALS associated genetic insults. These include both wild-type and mutant TDP43 and FUS, mutant SOD1, mutant *C9orf72*-derived dipeptide repeat polypeptides (DPRs) and Sigma-1 loss of function mutations [3, 7, 8, 18, 20, 29, 42, 47, 48, 50, 57]. VAPB and PTPIP51 are ER-mitochondria tethering proteins and for TDP43, FUS and *C9orf72* DPRs, this disruption to ER-mitochondria signaling involves breaking of the VAPB-PTPIP51 tethers [18, 47, 48]. This effect of wild-type and mutant TDP43 and FUS, and *C9orf72* DPRs on the VAPB-PTPIP51 interaction in turn disrupts ER-mitochondria contacts and IP3 receptor delivery of Ca^2+^ to mitochondria [18, 47, 48].

A primary function of ER-mitochondria signaling is to facilitate IP3 receptor mediated delivery of Ca^2+^ to mitochondria [6, 32, 39]. Thus, loss of VAPB and/or PTPIP51 inhibits whereas VAPB/PTPIP51 overexpression stimulates IP3 receptor delivery of Ca^2+^ to mitochondria [10, 16, 37, 47]. This delivery is essential for synaptic transmission and the VAPB-PTPIP51 tethers have been shown to regulate both pre- and post-synaptic function [17, 24].

Damage to synaptic function is a defining feature of FTD/ALS and this includes mutant TDP43 linked disease [4, 12]. Here we tested whether overexpression of VAPB or PTPIP51 to enhance ER-mitochondria signaling can correct TDP43 induced damage to ER-mitochondria Ca^2+^ exchange and linked synaptic function. Wild-type and four different FTD/ALS mutants of TDP43 including the two used in this study have been shown to disrupt the VAPB-PTPIP51 interaction and linked functions in neuronal cells and transgenic mice using quantitative assays, and expression of VAPB and PTPIP51 has been shown to stimulate ER-mitochondria signaling [47].

We studied VAPB or PTPIP51 expression alone as combined expression leads to a dramatic reorganisation of ER to envelop mitochondria, especially in higher level expressing cells [47]. This can lead to mitochondrial Ca^2+^ overload which is a signal for opening of the transition pore and apoptosis [6, 31, 32, 39]. We show that VAPB/PTPIP51 expression rescues mutant TDP43 damage to the IP3 receptor-VDAC1 channel, to IP3 receptor delivery of Ca^2+^ to mitochondria and to both synaptic vesicle release and dendritic spine numbers. This is the first demonstration that enhancing VAPB-PTPIP51 function to stimulate ER-mitochondria signaling can correct TDP43 linked damage to mitochondrial Ca^2+^ delivery and synaptic function.

We also show that UDCA, an FDA approved drug that is linked to FTD/ALS treatment, stimulates VAPB-PTPIP51 binding and corrects mutant TDP43 induced damage to both the VAPB-PTPIP51 interaction and ER-mitochondria Ca^2+^ delivery. Clinical trials are underway with UDCA and related compounds for ALS, Alzheimer’s disease and Parkinson’s disease but its protective method of action is unclear; it has been linked to protection against mitochondrial damage, ER stress, apoptosis, autophagy and inflammation in these neurodegenerative diseases [19, 38, 59]. These damaged functions are all regulated by ER-mitochondria signaling [6, 32, 39]. The mechanisms by which TDP43, FUS and mutant *C9orf72* DPRs disrupt VAPB-PTPIP51 binding are also not properly understood but involve activation of GSK3β [18, 47, 48]. We also show that UDCA inhibits GSK3β and rescues mutant TDP43 activation of GSK3β. Interestingly, others have presented evidence that UDCA inhibits GSK3β [11]. Thus, the beneficial effects of UDCA in FTD/ALS and other neurodegenerative diseases may involve at least in part, correction to damaged VAPB-PTPIP51 tethering and ER-mitochondria signaling via inhibition of GSK3β.

The mechanisms by which activation of GSK3β disrupts VAPB-PTPIP51 binding are not clear but one obvious hypothesis involves GSK3β phosphorylation of VAPB and/or PTPIP51; phosphorylation is a known mechanism for regulating protein-protein interactions. Both VAPB and PTPIP51 are heavily phosphorylated proteins (human VAPB contains 21 and PTPIP51 27 phosphorylation sites (see https://www.phosphosite.org/homeAction.action) but any GSK3β targeted residues have not as yet been identified. A proper testing of this hypothesis with therefore require the formal identification and subsequent experimental manipulation of such sites. Interestingly, whilst VAPB and PTPIP51 are enriched at ER-mitochondria contact sites, VAPB localises to all ER regions and PTPIP51 throughout the outer mitochondrial membrane [10, 47]. One possibility is that the inability of VAPB and PTPIP51 to interact is these non-contact regions is linked to their phosphorylation status. Over expression VAPB and PTPIP51 to enhance ER-mitochondria signaling such as we show here, may permit binding by increasing the levels of non-GSK3β phosphorylated VAPB and PTPIP51 that is competent to interact.

Whatever the precise mechanism, our findings reported here support the notion that targeting the VAPB-PTPIP51 tethers has therapeutic potential for FTD/ALS. Moreover, there is evidence that the VAPB-PTPIP51 interaction is also disrupted in Alzheimer’s disease and Parkinson’s disease; notably TDP43 pathology is a feature of both these diseases [28, 40, 45]. This includes VAPB-PTPIP51 disruption in induced pluripotent stem cell derived dopaminergic neurons from familial Parkinson’s disease patients carrying pathogenic *SNCA* (α-synuclein) triplications and in affected neurons in post-mortem Alzheimer’s disease brain [28, 40]. In these post-mortem Alzheimer’s disease cases, disruption to the VAPB-PTPIP51 interaction occurs early in disease arguing that it contributes to the pathogenic process in a primary fashion and is not just some epiphenomena [28]. Thus, correcting damaged VAPB-PTPIP51 tethers may also be beneficial for these other age-related neurodegenerative diseases.

## Conclusions

The studies described above support the notion that correcting damage to VAPB-PTPIP51 ER-mitochondria signaling may have therapeutic potential for FTD/ALS. We also provide evidence that UDCA, an FDA approved drug that along with analogues is in clinical trials for ALS, Parkinson’s disease and Alzheimer’s disease but whose precise therapeutic target is unclear, rescues TDP43 induced damage to the VAPB-PTPIP51 interaction.

## Data Availability

The datasets used and/or analysed during the current study are available from the corresponding authors on reasonable request.

## Abbreviations

ALS: amyotrophic lateral sclerosis
*C9orf72* DPRs: *C9orf72* dipeptide repeats
ER: endoplasmic reticulum
FTD: frontotemporal dementia
GSK3β: glycogen kinase-3β
IP3: inositol 1,4,5-trisphosphate
PTPIP51: protein tyrosine phosphatase interacting protein 51
TDP43: TAR DNA-binding protein-43
UDCA: ursodeoxycholic acid
VAPB: vesicle-associated membrane protein-associated protein B
VDAC1: voltage-dependent anion channel-1
